# Congenital Dislocation of the Knee: Idiopathic or Arthrogryposis?

**DOI:** 10.7759/cureus.21684

**Published:** 2022-01-28

**Authors:** Ricardo Barreto Mota, Nuno Rodrigues Santos, Rui Martins, Henrique Soares

**Affiliations:** 1 Neonatology, Centro Hospitalar Universitário de São João, Porto, PRT; 2 Pediatric Orthopedics, Centro Hospitalar Universitário de São João, Porto, PRT; 3 Obstetrics-Gynecology and Pediatrics, Faculdade de Medicina da Universidade do Porto, Porto, PRT

**Keywords:** prenatal diagnosis, term neonate, congenital dislocation of the knee, idiopathic clubfoot, arthrogryposis multiplex congenita

## Abstract

Fetal akinesia associated with fixed joints is a common cause for suspicion of arthrogryposis multiplex congenita, a severe condition with heterogeneous etiology. We present the case of a rarer but more benign condition, congenital knee dislocation.

The authors report the case of a 27-year-old woman medicated with levetiracetam for epilepsy whose prenatal ultrasound at 22 weeks of gestational age revealed bilateral clubfoot, permanent extension of the inferior limbs with internal knee rotation, normal amniotic fluid quantity, and fetal echocardiography. The remaining ultrasounds revealed similar results. Prenatal genetic testing revealed no pathological findings. The pregnancy was otherwise uneventful. A female newborn was delivered at 39 weeks by cesarean section, with no need for resuscitation. She presented with bilateral knee hyperextension and clubfoot, spontaneous movements, and normal mobility in all other joints. The remaining physical examination and brain and hip ultrasound on the second day of life were normal. These findings were compatible with idiopathic congenital dislocation of the knee (CDK). The patient was undergoing treatment with favorable evolution and adequate neurodevelopment, at the time of this report.

This case describes a diagnostic workup with the exclusion of severe syndromic pathologies, namely arthrogryposis. Despite the initial suspicion of arthrogryposis, a condition with a poor prognosis, this infant presented a more benign disease with favorable evolution.

## Introduction

Congenital dislocation of the knee (CDK) is a rare malformation (one in 100.000 neonates) [1], which is characterized by knee hyperextension and forward tibia displacement, affecting predominantly females [1]. Its diagnosis is established at birth based on physical examination in the immediate neonatal period followed by simple radiography. While its pathophysiology is not yet clearly understood, it is theorized that idiopathic cases are secondary to the mechanical molding of the fetus, causing joint contractures due to the insufficient in-utero space [2]. This condition may also be associated with hyperlaxity syndromes, such as Marfan or Ehlers-Danlos, Larsen syndrome, or meningomyelocele [1,3]. When joint contractures and hypomobility are detected in utero, suspicion for arthrogryposis multiplex congenita (AMC) must also arise.

## Case presentation

**Figure 1:**
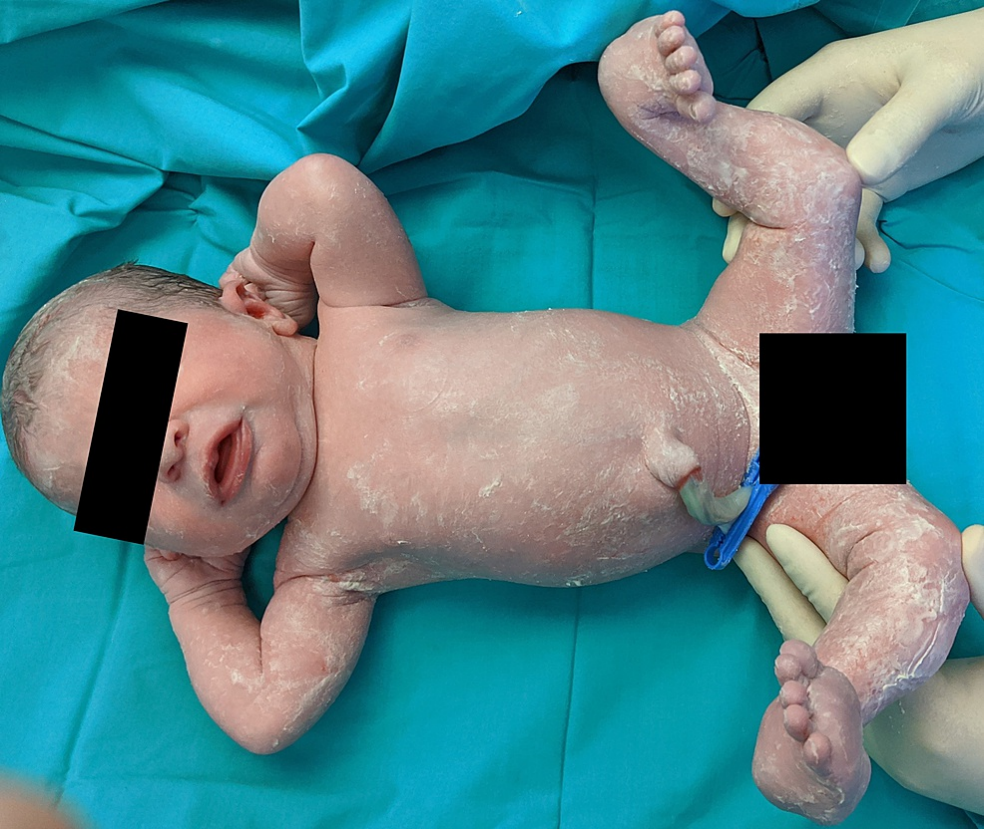
A term neonate with bilateral knee hyperextension and clubfoot

A prenatal ultrasound was performed in a 27-year-old woman medicated with levetiracetam for epilepsy at 17 weeks of gestational age, revealing bilateral clubfoot. Ultrasound at 22 weeks of gestational age revealed persistence of bilateral clubfoot, now associated with a permanent extension of the inferior limbs with internal knee rotation, reduced mobility, and cerebellum size in the lower normal limit. Amniotic fluid quantity and fetal echocardiography were normal. The remaining ultrasounds revealed similar results. Due to suspicion of arthrogryposis, amniocentesis was conducted at 18 weeks: Comparative genome hybridization was normal, aneuploidy search was negative, and a skeletal dysplasia genetic panel revealed no anomaly. The father was healthy and non-consanguineous. The pregnancy was otherwise uneventful. A female newborn was delivered at 39 weeks by cesarean section (in order to avoid birth lesions to the neonate), with no need for resuscitation and an adequate weight for gestational age. She presented with bilateral knee hyperextension and clubfoot (Figure 1), spontaneous movements, and normal mobility in all other joints.

The remaining physical examination and brain and hip ultrasound on the second day of life were normal. Lower limb radiographs were not performed. These findings were compatible with idiopathic grade III CDK.

## Discussion

The infant whose case we report presented bilateral knee hyperextension and clubfoot in utero as well as reduced fetal movements. These findings led to a suspicion of AMC, a vast heterogeneous group of disorders sharing multiple congenital contractures (MCC) and fetal akinesia as a common phenotype [4]. There are more than 400 disorders described with variable prognoses. They can be of intrinsic or extrinsic origin. Extrinsic factors may be secondary to intrauterine crowding (similar to idiopathic CDK) or to maternal diseases affecting the fetal central nervous system, with the former having a more favorable prognosis than the latter [5]. The intrinsic disorders have the worse prognosis, often being fatal in utero. The association between this pathology's incidence and the administration of levetiracetam during pregnancy is rarely reported in the literature.

Prenatal diagnosis is challenging, with in-utero detection between 26% and 66.7% described in the literature [5]. When conducting prenatal ultrasounds, joint contractures, abnormal limb position, brain anomalies, nuchal translucency, oligo or polyhydramnios, reduced fetal movements, or other malformations must raise suspicion for AMC [4]. These malformations may include clubfoot and congenital dislocation of the hip. These conditions may, however, also be present in idiopathic CDK [1], as was the case of the clubfoot described in our patient. When AMC is suspected, genetic testing must be considered, namely microarray, chromosomal disorders, deletions, or targeted molecular testing when there is a strong suspicion of a specific disorder [4]. Suspected and confirmed cases of AMC must be referred to a tertiary hospital in order to guarantee differentiated care to the newborn. A cesarean section must be conducted in order to avoid lesions in the neonate [4].

As previously described, in-utero genetic testing conducted on our patient was normal. Combined with the fact that the neonate presented normal mobility in the remaining joints and an otherwise normal physical examination, a diagnosis of congenital CDK was established. Its prognosis is usually favorable, and its response to treatment is mostly predicted by the joint’s mobility and stability as reported by Mehrafshan et al. [1] and Rampal et al. [6]. CDK is classified as type I when the knee is easily reducible and the joint remains stable in flexion. In type II CDK, the reduction with posteroanterior pressure is possible but reverts dislocation when pressure on the condyles is stopped. Our patient presented type III CDK, in which the knee is irreducible. The irreducible dislocation at birth, knee flexion < 50º, and absence of anterior skin groove appear to be risk factors associated with poorer response to surgery [6].

Our patient underwent Achilles tendon tenotomy in the first month of life, followed by serial cast immobilization. Due to unfavorable evolution, left knee quadricepsplasty was performed at three months of age and right knee quadricepsplasty at seven months of age. At the moment, she presents adequate neurodevelopment and favorable evolution. Postnatal genetic testing was, however, performed, with no results at the time of this report.

## Conclusions

We consider this to be a relevant case due to its rarity and antenatal diagnostic workup with the exclusion of syndromic pathologies. When in the presence of fetal akinesia, fixed joints, or more common musculoskeletal abnormalities such as club foot or hip dysplasia, utero genetic study may be relevant. Referral to a tertiary center and birth performed through a cesarian section must be pondered to avoid birth lesions to the neonate and to ensure early specialized evaluation, treatment, and follow-up.
